# Assessment of Potential Risk Factors and Skin Ultrasound Presentation Associated with Breast Cancer-Related Lymphedema in Long-Term Breast Cancer Survivors

**DOI:** 10.3390/diagnostics11081303

**Published:** 2021-07-21

**Authors:** Khairunnisa’ Md Yusof, Kelly A. Avery-Kiejda, Shafinah Ahmad Suhaimi, Najwa Ahmad Zamri, Muhammad Ehsan Fitri Rusli, Rozi Mahmud, Suraini Mohd Saini, Shahad Abdul Wahhab Ibraheem, Maha Abdullah, Rozita Rosli

**Affiliations:** 1 Department of Biomedical Sciences, Faculty of Medicine and Health Sciences, Universiti Putra Malaysia, Serdang, Selangor 43400, Malaysia; khairunnisabintimdyusof@uon.edu.au; 2 Hunter Medical Research Institute, New Lambton Heights, NSW 2305, Australia; kelly.kiejda@newcastle.edu.au; 3 School of Biomedical Sciences and Pharmacy, College of Medicine, Health and Wellbeing, The University of Newcastle, Newcastle, NSW 2308, Australia; 4 UPM-MAKNA Cancer Research Laboratory, Institute of Bioscience, Universiti Putra Malaysia, Selangor 43400, Malaysia; gs49066@student.upm.edu.my (S.A.S.); gs50830@student.upm.edu.my (N.A.Z.); gs48736@student.upm.edu.my (M.E.F.R.); 5 Centre for Diagnostic Nuclear Imaging, Universiti Putra Malaysia, Selangor 43400, Malaysia; rozi@upm.edu.my (R.M.); surainims@upm.edu.my (S.M.S.); 6 Department of Radiology, Faculty of Medicine and Health Sciences, Universiti Putra Malaysia, Selangor 43400, Malaysia; gs52320@student.upm.edu.my; 7 Department of Pathology, Faculty of Medicine and Health Sciences, Universiti Putra Malaysia, Selangor 43400, Malaysia

**Keywords:** BCRL, breast cancer, risk factors, self-reported, quality of life, arm swelling

## Abstract

Breast cancer has been reported to have the highest survival rate among various cancers. However, breast cancer survivors face several challenges following breast cancer treatment including breast cancer-related lymphedema (BCRL), sexual dysfunction, and psychological distress. This study aimed to investigate the potential risk factors of BCRL in long term breast cancer survivors. A total of 160 female breast cancer subjects were recruited on a voluntary basis and arm lymphedema was assessed through self-reporting of diagnosis, arm circumference measurement, and ultrasound examination. A total of 33/160 or 20.5% of the women developed BCRL with significantly higher scores for upper extremity disability (37.14 ± 18.90 vs. 20.08 ± 15.29, *p* < 0.001) and a lower score for quality of life (103.91 ± 21.80 vs. 115.49 ± 16.80, *p* = 0.009) as compared to non-lymphedema cases. Univariate analysis revealed that multiple surgeries (OR = 5.70, 95% CI: 1.21–26.8, *p* < 0.001), axillary lymph nodes excision (>10) (OR = 2.83, 95% CI: 0.94–8.11, *p* = 0.047), being overweight (≥25 kg/m^2^) (OR = 2.57, 95% CI: 1.04 – 6.38, *p* = 0.036), received fewer post-surgery rehabilitation treatment (OR = 2.37, 95% CI: 1.05–5.39, *p* = 0.036) and hypertension (OR = 2.38, 95% CI: 1.01–5.62, *p* = 0.043) were associated with an increased risk of BCRL. Meanwhile, multivariate analysis showed that multiple surgeries remained significant and elevated the likelihood of BCRL (OR = 5.83, 95% CI: 1.14–29.78, *p* = 0.034). Arm swelling was more prominent in the forearm area demonstrated by the highest difference of arm circumference measurement when compared to the upper arm (2.07 ± 2.48 vs. 1.34 ± 1.91 cm, *p* < 0.001). The total of skinfold thickness of the affected forearm was also significantly higher than the unaffected arms (*p* < 0.05) as evidenced by the ultrasound examination. The continuous search for risk factors in specific populations may facilitate the development of a standardized method to reduce the occurrence of BCRL and provide better management for breast cancer patients.

## 1. Introduction

Breast cancer is the most common cancer diagnosed worldwide with an estimated 2.3 million new cases in 2020 and it is more prevalent in less developed countries, such as Middle Africa and Eastern Asia compared to European countries [1]. In Malaysia, the National Cancer Registry [2] reported breast cancer (32.1%) as the leading cancer of female Malaysian residents, followed by colorectal (16.3%) and cervical cancer (7.7%). Despite its high incidence rate, breast cancer has a 5-year relative survival rate of 66.8%, the third highest among all cancers in females after corpus uteri (70.6%) and thyroid cancers (82.3%) [3].

The number of breast cancer survivors has improved over time due to early detection, improved treatments, and multi-disciplinary rehabilitation methods. However, the improved treatments also come with various late side effects such as arm or breast swelling (lymphedema), menopausal symptoms, sexual dysfunction, and psychological distress [4,5,6]. Approximately one in five breast cancer survivors will develop secondary lymphedema or breast cancer-related lymphedema (BCRL) in their lifetime [7].

BCRL often arises following the loss, obstruction, or blockage of lymphatic vessels due to cancer treatment including removal of lymph nodes or regional radiotherapy. It is characterized by abnormal lymphatic fluid retention and tissue swelling in one or both arms accompanied by discomfort, heaviness, functional dysfunction, and pain of the upper extremities; subsequently leading to decreased quality of life (QoL). BCRL has been reported to develop in 10–50% of women who undergo axillary lymph node dissection (ALND) and 5–20% of those who have sentinel lymph node biopsy (SLNB) [8,9,10]. The wide range of BCRL incidence is due to different methods applied in the diagnosis of lymphedema, the type of breast cancer treatment received, and the length of patient follow up in the studies [11,12]. Interestingly, BCRL does not occur immediately after surgery but often develops over time (starting with unresolved swelling after three months of surgery), suggesting multiple pathological causes that promote its progression. Most studies agree that impairment of the lymph flow is the first step of BCRL [13]. To date, ALND [7,9], regional radiotherapy [14,15], removal of more axillary lymph nodes [10,16], and higher body mass index (BMI) [17,18] are well-defined risk factors for developing BCRL. Other possible risk factors related to BCRL such as hypertension, chemotherapy, age, and genetic predisposition have yet to be investigated, or they have given inconsistent results in different studies [8,19,20].

At present, lymphedema that occurs at the upper quadrant extremities or hands is diagnosed by performing a series of tests that include physical examination, clinical history, measurement of arm volume, and examination of lymphatic structures through high-end imaging techniques such as computed topography. Physical examination such as arm circumference measurement, water displacement, bioimpedance analysis, and ultrasound examination are often carried out to incorporate differences between affected limbs from normal baseline [12]. Besides ruling out BCRL, these methods can also classify the stages and severity of lymphedema. The initial signs of BCRL are swelling and discomfort in the forearm area that disappears when lifting the affected area or by external compression [12,21]. Moderate to severe stages of BCRL consist of changes of the skin structure or appearance of *peau d’orange* (skin texture that resembles orange peel) that becomes bigger and is no longer relieved by lifting or external pressure [22]. The continuous expansion of fibrotic tissue and fat deposition affects the thickness of the skin and subcutaneous tissue, leading to the hardening of the skin. Previous studies have demonstrated that soft tissue changes and fat deposition in the affected forearm are associated with BCRL [23,24].

To our knowledge, studies on BCRL in the Asian region are lacking and this study is the first to address BCRL in Malaysian breast cancer survivors. Factors associated with BCRL vary among individuals, depending on the patient factors including age, BMI, stage of breast cancer, types of treatment, or comorbidities [18,25,26]. We aimed to investigate the potential risk factors associated with BCRL in a Malaysian cohort and to evaluate the skin structure of the affected arms. The search for factors associated with BCRL is crucial as early detection can facilitate more effective management and treatment to reduce the severity of lymphedema. 

## 2. Materials and Methods

### 2.1. Study Design and Participants

This matched case–control study was conducted in Malaysia from September 2018 until December 2019. The inclusion criteria for participants were as follows: (a) female aged 18 years or older; (b) Malaysian citizen; (c) diagnosed with breast cancer; (d) underwent unilateral breast cancer surgery at least three months prior to recruitment; (e) completed all chemotherapy and radiotherapy treatment; (f) no current evidence of cancer; and (g) able to provide informed consent. Meanwhile, the exclusion criteria involved (a) patients with other serious systemic illnesses (kidney failure, hepatic dysfunction, neurological, and psychological impairment); (b) history of corticosteroid use on the body for any reason; (c) abnormality or vascular disease in the upper extremities and (d) inability to provide informed consent.

**Figure 1:**
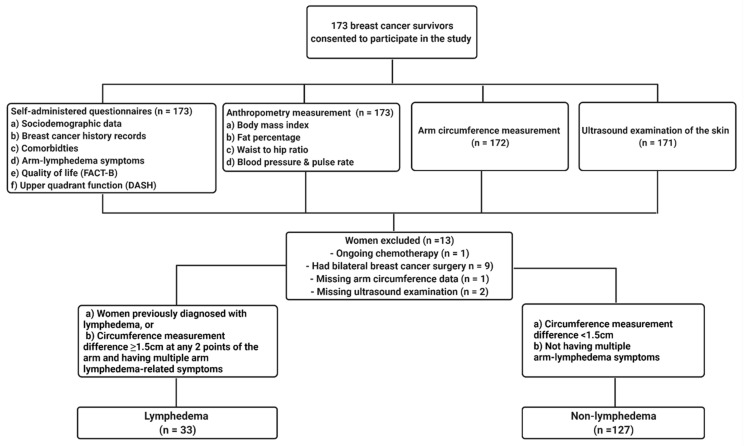
A flowchart of the recruitment and classification process of the breast cancer survivors in the study. FACT-B = Functional Assessment of Cancer Therapy—Breast. DASH = Disabilities of the Arm, Shoulder and Hand. CD = Circumference difference.

All participants gave informed consent. A set of questionnaires comprising of demographic and medical history, a QoL assessment, and upper quadrant function were self-administered by study participants. Participants were given the questionnaire verbally by the investigators, in cases where they had difficulties reading. Physical and anthropometry measurements (including weight, waist and hip circumference, fat percentage and blood pressure) were performed prior to measurement of arm circumference and ultrasound examination of the arms. Participants were provided with a minimal cash incentive for volunteering in the study. A total of 173 breast cancer survivors were initially recruited from eight different states in Peninsular Malaysia and 160 of them were eligible for the study (Figure 1).

### 2.2. Data Variables

#### 2.2.1. Anthropometrical Measurement and Questionnaires

Anthropometric measurements including blood pressure, pulse rate, weight, height, body mass index (BMI), waist to hip ratio (WHR) and fat percentage were recorded for each study participant. Blood pressure and pulse rate were recorded using blood pressure monitor (Microlife, Microlife Corporation, Taipei, Taiwan). A body composition monitor (Omron, OMRON Corporation, Osaka, Japan) was used to obtain weight and fat percentage values. BMI was calculated as weight (kilograms, kg) divided by height (meter, m^2^) and the categories were defined as normal (<25.0 kg/m^2^) and overweight (≥25.0 kg/m^2^). The WHR was measured by dividing the waist circumference (centimetres, cm) with hip circumference (cm) and classified as lower risk (<0.90) and higher risk (≥0.90) of abdominal obesity [27]. A bilingual (Malay and English) and self-administered questionnaire was provided to collect demographic, medical, and breast cancer information. The demographic information included current age, ethnicity, education level, employment status, number of children, income (<RM 3000/RM 3000 to RM 8000/>RM 8000), and marital status (single/married/widowed). The medical and breast cancer-related items included data such as age at first diagnosis, site of affected breast (left/right), breast biopsy (yes/no), staging (stage I/II/III/IV), receptor status (estrogen receptor (ER), progesterone receptor (PR), human epidermal growth factor receptor 2 (HER2) or triple negative breast cancer (TNBC), breast surgery (yes/no), date of surgery, type of breast surgery (left/right), modified radical mastectomy/mastectomy/lumpectomy (left/right/both sides), lymph node removal (yes/no), number of lymph nodes removed, details of treatment: chemotherapy/radiotherapy/hormonal therapy (yes/no), signs and symptoms of arm morbidities post-surgery (date of first start of swelling, part of swelling-forearm/upper arm) and post-surgery rehabilitation (physiotherapy/manual lymphatic drainage/massage/occupational therapy/fitness program/other). Additionally, comorbid medical conditions (diabetes/hypertension/cardiovascular disease/kidney disease/lung disease/infection/others), additional supplement intake, and family history of cancer (yes/no, mother’s side/father’s side/both sides) were also included.

The validated English [28] and Malay version [29] of *Functional Assessment of Cancer Therapy-Breast* or FACT-B obtained from FACIT.org was applied to access the QoL status of participants. The instrument consists of 37 items divided into five domains; seven items on physical (PWB), eight items on emotional (EWB), six items on social (SWB), and seven items on functional (FWB) well-being and 10 additional items on breast cancer scale (BCS) metrics. The scoring system of FACT-B is a Likert scale, which ranges from 0 to 4; 0—not at all, 1—a little bit, 2—somewhat, 3—quite a bit and 4—very much. The total score was calculated by adding all scores based on instructions in the manual (and reversing some of the items GP1-GP7, GE1, GE3-GE6, B1-B3, B5-B8 and B10). Higher scores indicate better QoL of an individual.

The DASH (Disability of Arm, Shoulder and Hand) questionnaire is a commonly used instrument to evaluate the upper extremity function of a patient when performing activities related to the upper limbs. It consists of 30 items with responses ranging from 1 to 5, out of which the individual rates their ability to perform specific activities in 25 items (1—no difficulty; 2—mild difficulty; 3—moderate difficulty; 4—severe difficulty; 5—unable). The remaining items in the instrument ask questions relating to the severity of symptoms, sleep interference, and feelings of confidence and usefulness (1—none; 2—mild; 3—moderate; 4—severe; 5—extreme). In the present study, the validated Malay version of DASH was employed for all participants [30] and the total score of all responses was converted to a single score ranging from 0 to 100, with higher scores indicating greater disability.

#### 2.2.2. Arm Lymphedema Assessment

The definition of arm lymphedema was first explained to the participants during the briefing, to avoid confusion with temporary swelling that occurs within three months after breast cancer surgery. Lymphedema occurring less than 12 months following breast cancer surgery was referred to as early-onset lymphedema, whereas swelling occurring more than one year after the surgery was considered late-onset lymphedema [31].

##### Self-Reporting of Diagnosis or Symptoms

Lymphedema cases that are diagnosed by a medical practitioner were reported within the questionnaire to classify the participants into the lymphedema or non-lymphedema sub-group. Additional self-reported symptoms and physical assessments were applied to characterize the participants’ arm morbidity. The self-reported soft tissue sensation symptoms of lymphedema included heaviness or tightness (yes/no), difficulty in finding shirts that fit (yes/no), pain at the arm area (yes/no) and swelling (yes/no).

##### Arm Circumference Measurement

A circumference measurement was performed by the same researcher (throughout the study) to identify the circumference difference (CD) between affected and unaffected arms of the participants. Measurements were taken on bilateral arms at four points; metacarpophalangeal joint, wrist, 10 cm distal to antecubital fossa of the elbow, and 15 cm proximal from the antecubital fossa to the lateral epicondyle of humerus, as visualized in Figure 1 [32]. Participants with self-reported arm morbidity and that had CD ≥ 1.5 cm at any two points of assessment were classified as having lymphedema [33].

##### Ultrasonographic Examination

**Figure 2:**
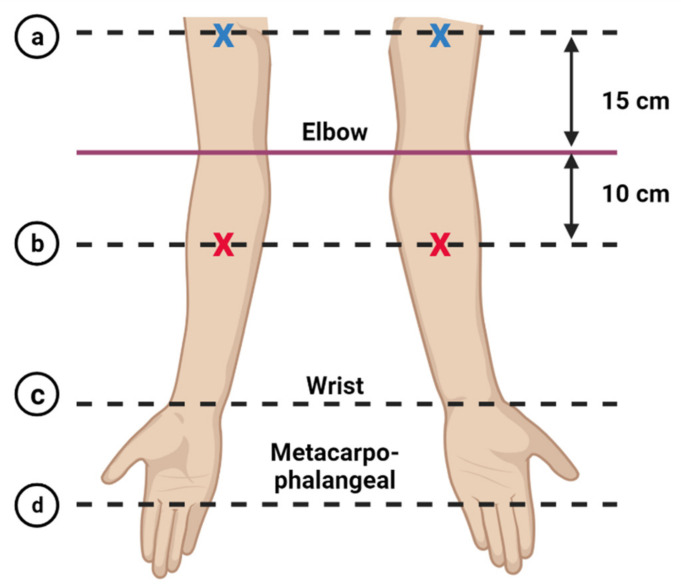
Points of the arm circumference measurement performed on the study participants. The four points are marked as (**a**) 15 cm from the antecubital fossa (anterior surface of the elbow) to the lateral epicondyle of humerus, (**b**) 10 cm distal to antecubital fossa of the arm, (**c**) the wrist and (**d**) the metacarpal-phalangeal joint. The ultrasound examination was performed on the mid-point of anterior surface of upper arm (blue X) and forearm (red X).

A trained researcher performed ultrasound imaging examinations using a linear probe with a 7.5 MHz transducer (model LSMUS-P0301-L75 Fujikin, Fujikin Co. Ltd., Osaka, Japan, connected to an Android system in a Samsung smartphone (Samsung Electronics Co. Ltd., Seoul, Korea) for image recording) at two sites of both arms; the anterior forearm at a point of 10 cm distal to the antecubital fossa and at 15 cm proximal from the antecubital fossa (Figure 2). The two points were chosen [33] and ultrasound images were recorded to measure the skinfold thickness (epidermis and dermis) and subcutaneous layers.

### 2.3. Statistical Analysis 

Descriptive statistics are expressed as the mean ± standard deviation (SD) for continuous variables and cross tabulation tables were applied for categorical variables. For normally distributed data, an independent *t*-test was performed to compare the mean between lymphedema and non-lymphedema groups, whereas a paired *t*-test was applied to compare the difference between affected and unaffected arms of lymphedema group participants. Non-parametric tests, such as the Mann–Whitney U and Wilcoxon signed rank test, were performed for non-normally distributed data. Spearman correlation test was performed to observe correlation exists between FACT-B and DASH scores, with lymphedema-related symptoms. 

For the assessment of lymphedema-related symptoms and risk factors, bivariate analysis (Pearson’s chi-square, Fisher’s exact or Likelihood-ratio) was applied for individual factors denoting *p* < 0.05 as statistically significant unless stated otherwise to find the odd ratios (OR). OR were calculated to illustrate the association between variables and lymphedema. A binary logistic regression was applied to identify factors associated with BCRL, using the enter stepwise method. All the significant and relevant variables from the bivariate analysis were classified as independent variables or predictors and lymphedema was set as a dependent variable. Predictors were revised for multicollinearity using a variance inflation factor (<3). 

The difference of arm circumference measurement was analyzed between unaffected and affected arms of participants in the lymphedema group. Finally, skinfold thickness measurements, including dermis and subcutaneous layer, were performed to compare differences between the affected and unaffected arms. All statistical analyses were performed using SPSS version 25 applying *p* < 0.05 as statistically significant. Graphpad PRISM 9.0 and Biorender.com software were used to generate graphs and diagrams of the findings.

## 3. Results

Of the 173 participants, 160 were eligible for inclusion in the study (with *n* = 7 having had secondary breast cancer). The mean age of study participants during recruitment was 51.04 ± 8.63 years, with a mean age following breast cancer diagnosis of 5.64 ± 4.34 years and a mean BMI of 27.88 ± 5.51. The study population consisted of three ethnic groups: Malay (*n* = 146, 91.3%), followed by Chinese (*n* = 11, 6.9%), and Indian/other (*n* = 3, 1.9%). The details of the demographic data are presented in Table 1.

### 3.1. Demographic, QoL and Upper Extremities Disability Analysis 

The presence of arm lymphedema was assessed in all 160 participants. Based on the self-reported symptoms and/or diagnosis, an individual with a CD of ≥1.5 cm between arms at any two points of measurement was classified into the lymphedema group (*n* = 33). Meanwhile, those with a CD of <1.5 cm at all points with less than two arm-lymphedema symptoms were classified as the non-lymphedema group (*n* = 127). There was a statistically significant difference between women with and without lymphedema for BMI, WHR values, number of children, FACT-B, and DASH scores between the two groups (Table 1). An extensive analysis was performed to determine the correlation between FACT-B, DASH, and the four main arm morbidities. A statistically significant and inverse correlation was found between FACT-B and DASH scores (*r* = −0.646, *p* < 0.001) (Table 2). All four symptoms were positively correlated with the DASH score (*p* < 0.01), ranging from a weak to moderate correlation. Apart from swelling of the arm, all arm symptoms were significantly correlated with FACT-B scores (*p* < 0.05). The details of the correlation of DASH, FACT-B, and the four arm-lymphedema symptoms are presented in Table 2.

### 3.2. Treatment-Related, Modifiable Factors and Arm Symptoms Associated with Lymphedema

Univariate analysis was performed by extracting the medical and breast cancer history of the study participants to identify individual factors associated with lymphedema (Table 3). Approximately 72.7% (*n* = 24) of women in the lymphedema group have fewer children as compared to those in the non-lymphedema group (48.0%, *n* = 61, OR = 2.89, 95% CI: 1.24–6.69, *p* = 0.011). Women in the lymphedema group had significantly higher BMI (78.8% of the women who developed lymphedema had a BMI ≥25 kg/m^2^) when compared to those who did not develop lymphedema (59.1% of the non-lymphedema group) (OR = 2.57, 95% CI: 1.04–6.38, *p* = 0.036). A higher WHR (≥0.90) was associated with an increased risk of lymphedema (OR = 2.37, 95% CI: 1.07–5.22, p = 0.030). The medical and treatment-related findings demonstrated that menopausal status (*p* = 0.256), age during breast cancer diagnosis (*p* = 0.449), breast biopsy (*p* = 0.298), side of breast affected (*p* = 0.730), stage of breast cancer diagnosis (*p* = 0.875), breast cancer receptors (*p* = 0.554), chemotherapy (*p* = 0.364), radiotherapy (*p* = 0.881) and anti-hormone therapy such as the estrogen receptor modulator (Tamoxifen) or aromatase inhibitor (Letrozole and Anastrazole) (*p* = 0.399) had no significant association with lymphedema. Additionally, family history of cancer (*p* = 0.982) and supplement intake (*p* = 0.890) were also not related to lymphedema.

Interestingly, there was a significant association with increased risk of lymphedema in women who had extensive surgeries (both mastectomy and lumpectomy) on the same side of breast (OR = 5.70, 95% CI: 1.21–26.8, *p* < 0.001) compared to those who had either mastectomy (OR = 1.24, 95% CI: 0.53–2.87, *p* = 0.618) or lumpectomy (OR = 0.72, 95% CI: 0.27–1.90, *p* = 0.505). Although there were missing data for the total number of lymph nodes removed (*n* = 40, 25.0%), excision of more than 10 lymph nodes was significantly more common in the lymphedema group (OR = 2.83, 95% CI: 0.94–8.11, *p* = 0.047). Women who received none or only had one type of post-surgery rehabilitation treatment were at increased risk of lymphedema (OR = 2.37, 95% CI: 1.05–5.39, *p* = 0.036). Hypertension showed a significant association with BCRL (33.3% in lymphedema group and 17.3% in non-lymphedema group, OR = 2.38, 95% CI: 1.01–5.62, *p* = 0.043). There was no significant association between the two groups for other comorbid medical conditions including cardiovascular disease, lung or kidney disease, or infection (*p* > 0.05). 

Two out of four main symptoms were found to have a significant association with lymphedema including hardness and difficulties in finding shirts that fit (OR = 4.63, 95% CI: 2.05–10.49, *p* = 0.001) and swelling of the arms (OR = 73.2; 95% CI: 16.3–329.6, *p* = 0.001). No significant difference was found for heaviness or tightness of the chest (*p* = 0.916) and pain in the arm area (*p* = 0.089) (Table 3).

A binary logistic regression analysis was performed on relevant individual factors that were significantly associated with lymphedema, including the BMI, having extensive surgeries, and hypertension, and received less post-surgery rehabilitation. No multicollinearity was found between the variables. Several factors that were excluded such as total number of lymph nodes excised due to 25.0% of missing data (lymphedema, *n* = 5, non-lymphedema, *n* = 35) and WHR as the cut-off value for higher risk of metabolic syndrome exceeded normal value (≥0.90) in both groups. The analysis showed that higher BMI, hypertension, and post-surgery rehabilitation treatment were slightly attenuated (*p* > 0.05), whilst extensive surgeries (lumpectomy and mastectomy) maintained its statistical significance in the model (Table 4). Details of the analysis are presented in Table 4.

### 3.3. Objective Assessment of Breast Cancer Survivors with Lymphedema

Among the 33 participants in the lymphedema group, approximately 48.5% (*n* = 16) of the women developed lymphedema at early-onset whereas 51.5% (*n* = 17) had late-onset lymphedema. The characteristics of women who developed lymphedema with early or late-onset are shown in Table 5. The FACT-B scores were significantly lower for those who had early-onset lymphedema when compared to those who had late-onset lymphedema (95.9 ± 21.7 vs. 111.4 ± 19.6, *p* = 0.040). No significant difference was found between any of the treatment-related factors, with the exception of the number of lymph nodes removed. Study participants who developed early-onset lymphedema had more lymph nodes removed (*n* = 13, 19.0 ± 6.5) as compared to those who developed late-onset lymphedema (*n* = 15, 12.2 ± 8.8, *p* = 0.028). Additionally, the odds of having early-onset lymphedema were nine times higher in diabetic patients when compared to those who were not diabetic (OR = 9.60, 95% CI: 1.00–91.96, *p* = 0.039).

#### 3.3.1. Arm Circumference Measurement

**Figure 3:**
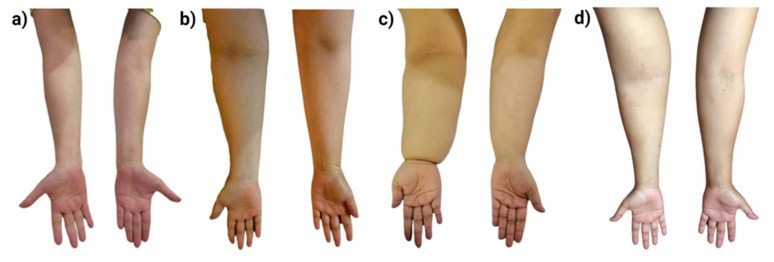
Differences in unaffected and affected arms of breast cancer subjects diagnosed with existing arm lymphedema. Figure (**a**) and (**b**) display mild stage of lymphedema, manifested by significant difference at the forearm area when compared to the other sites of the affected arms. Meanwhile, figure (**c**) and (**d**) show moderate to severe stage of lymphedema, characterized by swelling in major sites of the arms including metacarpal-phalangeal joint, wrist, forearm, and upper arm area.

The arm circumference measurement was taken at four points and compared between unaffected and affected arms of women in the lymphedema group. It was found that the CD of the metacarpo-phalangeal, wrist, forearm and upper arm were significantly higher on the affected arm (0.44 ± 0.96, 0.82 ± 1.43, 2.07 ± 2.48 and 1.34 ± 1.91, *p* < 0.05). Details on the measurement are presented in Table 6. Images of difference in the measurement of arm circumference between the affected and unaffected arms are shown in Figure 3. Figure 3a,b show mild stage lymphedema whereas Figure 3c,d demonstrate moderate to severe stage of lymphedema.

#### 3.3.2. Ultrasound Examination Analysis

**Figure 4:**
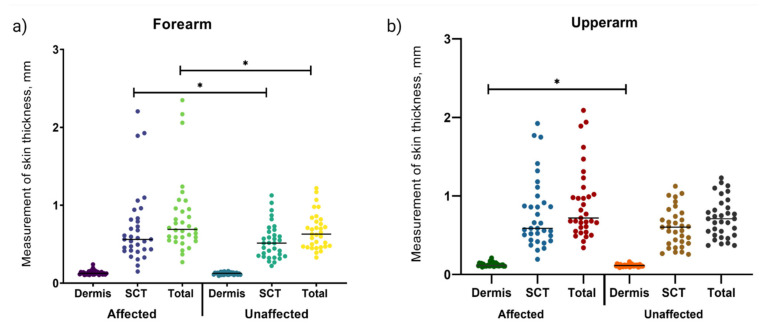
Comparison of skin thickness measurements of the dermis and subcutaneous layers as well as the total (sum of dermis and subcutaneous layer) between affected and unaffected arms evaluated in women with lymphedema (*n* = 33). (**a**) Measurement of the forearm area of both affected and unaffected arms. A significant difference was found on the subcutaneous layer and total measurement between arms. (**b**) Measurement of the upper arm area, with significant difference on the dermis layer between affected and unaffected arms. *p value < 0.05 was calculated from Wilcoxon-signed rank analysis. Subcutaneous SCT.

As shown in Figure 4, the measurements of skinfold thickness at four points between the affected and unaffected arm displayed a significant difference in the subcutaneous (*p* = 0.048) and total thickness (*p* = 0.027) of the forearm areas (Figure 4a) and dermis layer of the upper arm area (*p* = 0.030) (Figure 4b). No significant differences were found in the forearm measurements between the unaffected and affected arm for the dermis (Figure 4a) or in the measurements of the upper arm in the subcutaneous layer or the total thickness (*p* > 0.05) (Figure 4b). 

**Figure 5:**
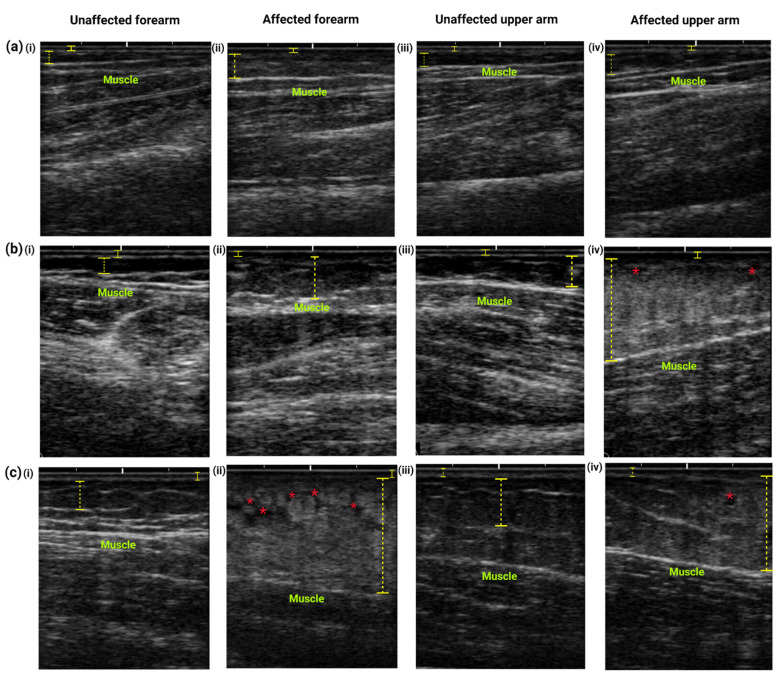
Typical images of ultrasound examination of the (**i**) unaffected forearm, (**ii**) affected forearms, (**iii**) unaffected upper arm and (**iv**) affected upper arm performed on women with (**a**) mild and (**b,c**) moderate to severe stage lymphedema. The structures of the skin measured included the dermis and epidermis layers (yellow straight lines) and the subcutaneous layer (yellow discontinuous lines). The images of the unaffected forearm and upper arm display typical skin structure with normal anatomical structures of the dermis, subcutaneous, and muscle layers. Mild lymphedema is characterized by thickening of subcutaneous layer at the (**a**(**ii**)) forearm and (**a**(**iv**)) upper arm. In contrast, tissue changes occur mainly at the subcutaneous area, as lymphedema progresses. As demonstrated in (**b,c**)**,** tissue thickness increased in the affected arms (**b**(**ii,iv**) and **c**(**ii,iv**)) due to accumulation of fluid in the subcutaneous tissue (indicated by hyperechogenic tissue), which is associated with higher stages of lymphedema. A blurring interface between the subcutaneous and dermis layers is also observed in the affected arms (**b**(**ii,iv**) and **c**(**ii,iv**)). Prominent subcutaneous edema and fat lobules give the cobblestone or lattice pattern (red asterisks), indicating soft tissue changes that often occur in the subcutaneous layer.

The ultrasound images of the affected and unaffected arms of individuals with different stages of lymphedema display different patterns of tissue structures mainly within the subcutaneous areas (Figure 5). Figure 5a demonstrates thicker subcutaneous layer of the forearm (Figure 5a(ii)) and upper arm (Figure 5a(iv)) area, with limited thickening of the dermis layer when compared to the unaffected arms (Figure 5a(i,iv)). Figure 5b,c represent skin structures of the moderate to severe stage lymphedema. The dermis layer of the unaffected forearm (Figure 5b(i),c(i)) and upper arm (Figure 5b(iii),c(iii)) are hyperechoic (brighter color) whereas the affected forearm (Figure 5b(ii),c(ii)) and upper arm (Figure 5b(iv),c(iv)) show a more hypoechogenic structure (black color), representing typical edema or water retention. Meanwhile, the unaffected forearm and upper arms manifested less echogenicity and a thinner subcutaneous layer. White streaks are prominent in the unaffected area indicating the clear muscle compartment. The subcutaneous layer of the affected forearm and upper arm are hyperechogenic (bright color), indicating the accumulation and penetration of fat and fibrous tissue in the area (cobblestone structure). 

## 4. Discussion

BCRL is a progressive condition that affects the upper extremity function and that can cause detrimental effects on the quality of life of an individual. It is unclear why some breast cancer patients develop lymphedema, while those who received identical treatment do not. Based on the literature, the risk factors associated with BCRL are divided into treatment-related (i.e., types of surgery, number of dissected lymph nodes, regional radiotherapy) and modifiable risk factors (comorbidities, obesity, and lower physical activity).

In this study, one in five breast cancer survivors (21.5%) who have undergone unilateral breast cancer surgery developed BCRL. Note that this is the first study to address the occurrence of BCRL in the Malaysian population, and the percentage of BCRL is in line with most reported studies [7,21], despite the different methods applied in the study to evaluate lymphedema status. Our findings indicate that having fewer than three children may increase the risk of developing BCRL two-fold. Considering that more than 50% of the study participants are homemakers, frequent use or movement of the treated side to care for their family, such as preparing meals and doing house chores could not be avoided. Our results also support the theory that those who have a sufficient level of physical activity have better upper-body function [34,35].

BCRL is considered one of the most distressing side effects from breast cancer treatment to some patients as it can cause physical and functional dysfunction, psychological disturbance, as well as an alteration of body image. The assessment of QoL is important to provide information on the long term-impact of cancer treatment and to provide better management for affected individuals [36]. The present study revealed a significantly lower score for FACT-B in the lymphedema group compared to the non-lymphedema group, suggesting BCRL decreased the QoL of breast cancer survivors. Similar findings were observed in previous studies where QoL scores were significantly lower in BCRL patients when compared to non-BCRL participants or the normal group [37,38,39,40].

The present study also revealed higher BMI (≥25 kg/m^2^) as a factor associated with a two-fold increased risk of developing BCRL, as shown in univariate and only slightly attenuated in multivariate analysis. According to the WHO classification for the Asian population, a BMI of ≥25 kg/m^2^ is considered overweight, while ≥30 kg/m^2^ is obese I [41]. In comparison, the average BMI for our non-lymphedema group was classified as overweight (27.3 kg/m^2^), while the lymphedema group was classified as obese (30.0 kg/m^2^). Higher BMI and obesity have been well documented as novel and independent risk factors of developing BCRL in many studies [17,21,25,42]. Obesity has been well documented as a novel risk factor associated with BCRL, due to fat hypertrophy in lymphedematous tissue and fibrosis. The lymphatic vessels in the axillary area are usually disrupted after breast cancer surgery or lymph node dissection and leads to the accumulation of interstitial fluid, before eventually inducing adipose tissue differentiation and local fat deposition [43,44].

WHR is considered complementary to BMI to assess the risk of developing obesity-related morbidities. Note that Asians have a smaller body size than Westerners, therefore, there is a higher frequency of localized obesity and fat accumulation rather than whole-body obesity [45]. A study by Yoon et al. reported a correlation between the severity of arm lymphedema with abdominal obesity. In contrast, our study population showed both groups are at increased risk of developing metabolic syndrome as the WHR exceeded the reference value for Asian women, >0.80.

Radical mastectomy, regional radiotherapy, chemotherapy drugs such as taxane or docetaxel, and ALND have been well studied as treatment-related factors associated with BCRL [10,46,47]. Our present findings demonstrated that mastectomy, radiotherapy, or chemotherapy on its own did not increase the risk of BCRL. However, multiple surgeries (both mastectomy and lumpectomy) on the same breast were found to be associated with BCRL. The odds of BCRL in women who had multiple surgeries increased by 5.7 to 5.9, as compared to those who had either mastectomy or lumpectomy alone. Multiple surgeries may be linked with aggressive systemic therapies and greater extent of axillary lymph node clearance. The findings are in line with a study by Nguyen and colleagues, which reported that BCRL is a consequence of not a single cause, but multimodal treatments including the extent of axillary surgery, radiotherapy, and taxane-based chemotherapy [9]. Furthermore, our findings revealed that excision of 10 or more lymph nodes increased the likelihood of BCRL by three-fold. These findings are in line with Kim et al. who reported the incidence rates of BCRL in patients with more than 10 lymph nodes removed were 19% and significantly higher than those who had less than 10 nodes removed [48]. Killbreath et al. also reported the association of BCRL with the number of lymph nodes excised where patients who had more than five lymph nodes removed had a 15% increased rate of BCRL compared to patients with fewer nodes removed [10]. 

Other comorbidities, such as hypertension and diabetes, were also found to contribute to BCRL development. Our study revealed a positive association between hypertension and BCRL, although the association was diminished in the multivariate analysis. During hypertension, an elevated hydrostatic pressure in blood vessels increases capillary filtration between lymphatic and blood vessels. The continuous vicious cycle between the systems increases pressure and lymphatic flow, inducing lymphatic vessel hyperplasia and subsequently facilitating leakage of the lymph into the tissues, leading to swelling of the affected areas [49,50]. The association of hypertension as a risk factor affecting BCRL has yet to be elucidated as evidence on the correlation between hypertension and BCRL has been inconsistent, with some studies reporting a positive association [20,49,51] and others reporting no association [42,52].

Studies on the long-term survivorship of breast cancer found a positive association of diabetes mellitus with arm morbidities [53,54]. Our study revealed that diabetic patients had a nine-fold increased likelihood of developing early-onset lymphedema as compared to those who were not. It was reported that breast cancer patients with diabetes undergoing mastectomy or extensive tissue dissection are challenged with delayed wound healing due to disruption of the local blood supply and tissue hypoxia [55]. Therefore, we hypothesized that the occurrence of BCRL in diabetic patients was increased due to the delayed wound healing caused by breast surgery and the increased arm morbidity associated with these individuals. Moreover, a study on the Latino breast cancer population found post-operative participants with diabetes mellitus were likely to report more lymphedema symptoms such as swelling [54].

Since there is no molecular-based therapy to treat BCRL to date, the standard management or treatment of BCRL applied to reduce severity and the degree of arm-morbidity symptoms include manual lymphatic drainage (MLD), compression garments, physiotherapy, remedial exercise or fitness programs, massages, skin care routines, and surgical treatment for advanced stage lymphedema. Based on our findings from the univariate analysis, participants who had combined physical therapy or rehabilitation (i.e., manual lymphatic drainage, physiotherapy, fitness program) had a 2.4-fold reduced rate of BCRL. It is well documented that combined physical therapy is more effective in reducing the severity of lymphedema as it can improve the lymph circulation through repeated contraction and muscle relaxation [56]. A one-year study on complete digestive therapy (CDT) combined with self-administered CDT on 41 breast cancer patients showed an improved Numerical Pain Rating Scale (NPRS) score and self-administered CDT for six months significantly decreased the excess limb volume by 8% in the experimental group [57]. Besides MLD and CDT, a combined resistance and aerobic exercise have been proven to have a positive effect on BCRL as reported in several studies [56,58,59,60].

The present study combined self-reported questionnaires (DASH, symptoms like heaviness, swelling of the arm, pain at any part of the upper extremities and difficulties in finding shirts that fit) and arm circumference measurements to discriminate women who may have lymphedema. Self-reported questionnaires are useful to observe early lymphedema-related signs and provide the capacity to identify those likely to be disease-free [61]. Moreover, precise BCRL incidence is hard to be identified due to non-standardized diagnostic methods, hence self-reporting symptoms facilitates further clinical investigations for lymphedema. In our study, swelling of the arm and difficulties finding shirts that fit were significantly associated with the lymphedema group. Arm swelling has been well documented as a major physical sign of lymphedema. Persistent swelling of the arm caused women to experience difficulties in finding shirts that fit both hands due to the different size of the arms or restricted range of movement of the upper extremities. Meanwhile, heaviness or tightness may not necessarily be experienced until increased limb volume occurs and lymphedema worsens [61]. Our study revealed that the DASH scores were higher in the lymphedema group and negatively correlated with the QoL scores. The DASH questionnaire has been extensively applied to evaluate shoulder dysfunction or lymphedema following cancer treatment in breast cancer populations [62,63,64]. Greater disability of the arms negatively affected the physical functions and QoL of affected individuals as reported in many studies of breast cancer patients [65,66,67,68].

Arm circumference measurement using tape has been widely used to monitor the difference between arms and is inexpensive and easy to use. In fact, self-monitoring using tape measurement helps make the individual aware of any difference related to their limbs [69,70]. Our study revealed that the distal forearm areas, including metacarpo-phalangeal and wrist, were minimally affected by the swelling, unlike the proximal forearm and distal upper arm areas. Moreover, the largest CD was found within the forearm. These findings may result from several factors. Firstly, accumulation of fluid predominantly confined at the proximal forearm area minimally affects the distal area of the forearms (hands) [71,72]. This could be due to anatomical differences of epifascial vessels (skin and subcutis drainage) as edema tends to occur at the subcutis and skin compartment that are prominent in the forearm and upper arm areas. Moreover, it was reported that there is dermal backflow of lymphatic drainage of the hand of the swollen arms to the distal forearm [73,74]. Secondly, the accumulation of fluid is gravity dependent and unlike the forearm, the upper arm area has Mascagni’s cephalic path which provides better circulation to evacuate fluid at the upper extremities [75]. Third, the study population includes mild to severe lymphedema, which explains inconsistent measurement of the arms between the participants.

The most common criterion to diagnose lymphedema from circumference measurements is to have equal or greater than 2 cm inter-limb difference at any single location as a cut-off value [12]. However, in our study and others, this value seemed too large to identify milder cases of lymphedema [70] and may not accurately generalize to all populations with different body size distributions, including Asians who typically have a smaller body size than Western individuals. According to Petrek et al., a difference of less than 1.27 cm was considered mild lymphedema when accompanied by self-reported swelling or heaviness, 1.27 cm to 5.08 cm was considered moderate and greater than 5.08 cm was defined as having severe lymphedema [76]. Similarly, Can et al. classified those who had an arm difference of <3 cm as mild lymphedema, 3–5 cm as moderate, and greater than 5 cm as severe lymphedema [77]. Collectively, our results suggest that there is no specific cut-off value that could fit the entire study population, as the difference in arm circumference may be influenced by the irregular shape of the arms and body size of affected individuals.

Skin ultrasound examination is a standard diagnostic method to evaluate edema and tissue changes. The application of ultrasound is effective to evaluate severity, composition of epifascial, subfascial, fibrosis, and fluid accumulation over time [12]. The comparison of echogenicity facilitates the assessment of lymphedema more precisely and is cost effective when compared to other imaging procedures. Our present study revealed greater thickness of the subcutaneous tissue of the forearm with limited skin thickening of the affected arms when compared to unaffected arms. However, the dermis layer of the affected arms was hypo-echogenic, indicating typical water accumulation in edema patients.

These findings are supported by van der Veen et al. who reported hypoechoic dermis of the affected arms [75]. The arm circumference measurements in our study (that demonstrated greater CD at the forearm area) were reflected by subcutaneous thickness measured by ultrasonography. In comparison to the affected upper arm, the echogenicity of the subcutaneous layer was higher in the affected forearm, indicating the presence of fat lobules and fibrous tissue in the area characterized by a cobblestone or lattice-like pattern. Similarly, previous studies have reported a positive correlation between subcutaneous ultrasound hyper-echogenicity with increased volume or arm circumference of the lymphedematous forearms [24,78]. The disruption of lymphatic vessels induces inflammation, which later promotes the production of extracellular matrix components such as collagen, elastin and fibronectin. The continuous production of extracellular matrix components and fat deposition resulted in dense composition at the subcutaneous area [79].

There are several limitations to our study. Given that we applied a case–control study design, the number of BCRL women in our study did not reflect the prevalence of BCRL in our population. Therefore, we could not determine the cause-effect relationship. Reporting of variables such as pitting edema, the number of excised axillary lymph nodes, type of breast cancer receptors, radiotherapy and chemotherapy drugs by participants in this study were limited. Due to this, our results may be skewed because of the missing data for these variables. Although there may be missing cases of subclinical lymphedema, self-reporting symptoms were supported by arm circumference measurements and ultrasonography data. We did not factor in hand dominance of the participants, which may affect the use of the upper extremities and the ability of the participants to perform certain physical activities, assessed in DASH and FACT-B questionnaires. Furthermore, applying a low frequency transducer for ultrasonography may facilitate the evaluation of tissue changes in the skin and subcutaneous tissue, but staging of lymphedema can only be assessed using a high frequency probe (15–20 MHz).

## 5. Conclusions

The findings of this study suggest that BCRL incidence is affected by both treatment (i.e., multiple surgeries, more axillary lymph nodes excision, and lacking post-operative rehabilitation) and patient-related factors (obesity, hypertension, or diabetes). The combined application of screening and assessment methods such as self-reported symptoms and arm circumference measurement facilitated the classification of breast cancer survivors into the lymphedema group. Continuous and extensive studies on BCRL factors are important for the development of a standardized method to reduce BCRL incidence, providing better management and treatment of this complication for breast cancer survivors.

## Figures and Tables

**Table 1:** Baseline characteristics of breast cancer survivors with and without lymphedema reported at recruitment (N = 160).

Characteristics	Lymphedema (*n* = 33)	Non-Lymphedema (*n* = 127)	*p* Value
Age ^a^	51.73 ± 8.15	50.87 ± 8.78	0.597
Age at diagnosis (years) ^a^	46.42 ± 7.92	45.23 ± 8.35	0.449
Years after diagnosis ^b^	5.30 ± 4.10	5.72 ± 4.40	0.220
Body mass index (kg/m^2^) ^b^	30.03 ± 5.05	27.32 ± 5.49	0.005 **
Waist-to-hip ratio ^a^	0.91 ± 0.06	0.88 ± 0.06	0.048 *
Systolic blood pressure (mm/Hg), (*n* = 126)	129.72 ± 20.58	126.13 ± 18.14	0.373
Diastolic blood pressure (mm/Hg), (*n* = 126)	80.56 ± 12.16	80.48 ± 13.22	0.975
Pulse rate (per minute), (*n* = 126)	84.41 ± 14.25	80.56 ± 12.26	0.169
QoL scores (FACT-B) ^b^	103.91 ± 21.80	115.49 ± 16.80	0.009 **
Arm disability scores (DASH) ^b^	37.14 ± 18.90	20.08 ± 15.29	<0.001 ***
Ethnicity ^c^			
• Malay	29 (87.8%)	117 (92.1%)	0.237
• Chinese	4 (12.1%)	7 (5.5%)	
• Indian/other	0 (0.0%)	3 (2.4%)	
Educational status ^d^			
• Primary & secondary school	17 (51.5%)	81 (63.8%)	0.198
• College & university	16 (48.5%)	46 (36.2%)	
Employment status ^d^			
• Professional	7 (21.2%)	21 (16.5%)	0.182
• Service/freelance	4 (12.1%)	28 (22.0%)	
• Homemaker	21 (63.6%)	64 (50.4%)	
• Retired	1 (3.0%)	14 (11.0%)	
• Missing ^e^	0 (0.0%)	1 (0.1%)	
Monthly income (RM) ^d^			
• <3000	25 (75.8%)	87 (68.5%)	0.588
• 3000–8000	6 (18.2%)	34 (26.8%)	
• >8000	2 (0.1%)	6 (4.7%)	
Marital status ^d^			
• Married	30 (90.9%)	119 (93.7%)	0.572
• Single/divorced	3 (9.1%)	8 (6.3%)	
Number of children ^d^			
• None	7 (21.2%)	12 (9.5%)	0.023 *
• 1–3 children	17 (51.6%)	49 (38.6%)	
• More than 3 children	9 (27.2%)	66 (51.9%)	
Menopausal status ^d^			
• Post-menopausal	25 (75.8%)	83 (65.6%)	0.256
• Pre-menopausal/unknown	8 (24.2%)	44 (34.6%)	

QoL = quality of life; FACT-B = Functional Assessment of Cancer Therapy—Breast; DASH = Disabilities of Arm, Hand, and Shoulder; RM = Malaysian ringgit. ^a^ *p* value of differences between means were calculated using independent Student’s *t*-test, ^b^ *p* value of differences between means were calculated from Mann–Whitney U test, ^c^ Test of association using Likelihood ratio, ^d^ Test of association using the Pearson’s chi-squared test, ^e^ *p* value does not include missing info. Statistically significant values are indicated by * *p* < 0.05, ** *p* < 0.01, and *** *p* < 0.001.

**Table 2:** Correlation between the FACT-B, DASH, and arm morbidities.

Domain	DASH	Heaviness/Tightness	Difficulties in Finding Shirt That Fits	Pain in the Arm	Swelling of the Arm
DASH	1	0.192 *	0.388 **	0.309 **	0.316 **
FACT-B	−0.646 **	−0.179 *	−0.342 **	−0.316 **	−0.123

Spearman correlation test, * *p* value < 0.05 and ** *p* value < 0.01. FACT-B = Functional Assessment of Cancer Therapy—Breast; DASH = Disabilities of Arm, Hand, and Shoulder.

**Table 3:** Comparison of medical characteristics, treatment-related factors, and arm morbidities of the study population.

Characteristics	Lymphedema (*n* = 33)	Non-Lymphedema (*n* = 127)	OR [95% CI]	*p* Value
Number of children ^a^				
• 0 to 3 children	24 (72.7%)	61 (48.0%)	2.89 [1.24–6.69]	0.011 *
• More than 3 children	9 (27.3%)	66 (52.0%)		
Body mass index, (kg/m^2^) ^a^				
• ≥25	26 (78.8%)	75 (5915%)	2.57 [1.04–6.38]	0.036 *
• <25	7 (21.2%)	52 (40.9%)		
Waist-to-hip ratio ^a^				
• ≥0.90	21 (63.6%)	54 (42.5%)	2.37 [1.07–5.22]	0.030 *
• <0.90	12 (36.4%)	73 (57.5%)		
Affected side ^a^				
• Left	18 (54.5%	62 (48.8%)	0.87 [0.72–0.85]	0.730
• Right	15 (45.5%)	65 (51.2%)		
Breast biopsy ^b^				
• Yes	33 (100.0%)	121 (95.3%)	0.79 [0.72–0.85]	0.298
• No	0 (0.0%)	4 (3.1%)		
• Missing/unknown ^c^	0 (0.0%)	2 (1.6%)		
Tumor stage ^d^				
• I	7 (21.2%)	28 (22.0%)	0.96 [0.61–1.53]	0.875
• II	17 (51.5%)	63 (49.7%)		
• III	6 (18.2%)	28 (22.0%)		
• IV	3 (9.1%)	8 (6.3%)		
Receptor status ^a^				
• Positive-receptor breast cancer	28 (84.8%)	99 (77.9%)	1.41 [0.45–4.48]	0.554
• (ER/PR/HER2)				
• Triple negative breast cancer	4 (12.1%)	20 (15.7%)		
• Missing/unknown ^c^	1 (3.0%)	8 (6.4%)		
**Type of breast surgery ^a^**				
Lumpectomy				
• Yes	6 (18.2%)	30 (23.6%)	0.72 [0.27–1.90]	0.505
• No	27 (81.8%)	97 (76.4%)		
Mastectomy				
• Yes	23 (69.7%)	94 (74.0%)	0.87 [0.35–1.87]	0.618
• No	10 (30.3%)	33 (26.0%)		
Lumpectomy & mastectomy				
• Yes	4 (12.1%)	3 (2.7%)	5.70 [1.21–26.8]	0.015 *
• No	29 (87.9%)	124 (97.3%)		
Axillary lymph nodes excision				
• Yes	33 (100.0%)	121 (95.3%)	0.79 [0.72–0.85]	0.298
• No	0 (0.0%)	4 (3.1%)		
• Missing/unknown ^c^	0 (0.0%)	2 (1.6%)		
No. of lymph nodes removed ^a^				
• ≥ 10	23 (69.6%)	57 (44.8%)	2.83 [0.98–8.12]	0.047 *
• < 10	5 (15.2%)	35 (27.6%)		
• Missing/unknown ^c^	5 (15.2%)	35 (27.6%)		
**Breast cancer treatment ^a^**				
Chemotherapy				
• Yes	25 (75.8%)	105 (82.6%)	0.66 [0.26–1.64]	0.364
• No	8 (24.2%)	22 (17.2%)		
Radiotherapy				
• Yes	24 (72.7%)	94 (74.0%)	0.94 [0.39–2.22]	0.881
• No	9 (27.3%)	33 (25.9%)		
Hormonal therapy				
• Yes	27 (81.8%)	82 (64.6%)	1.52 [0.57–4.00]	0.399
• No	6 (18.2%)	43 (33.8%)		
**Post-surgery rehabilitation ^a^**				
• One-type treatment	22 (69.7%)	62 (48.8%)	2.37 [1.05–5.39]	0.036 *
• Taking two or more treatment	11 (30.3%)	64 (50.4%)		
• Missing/unknown ^c^	0 (0.0%)	1 (0.8%)		
**Comorbidities ^a^**				
Hypertension				
• Yes	11 (33.3%)	22 (17.3%)	2.38 [1.01–5.62]	0.043 *
• No	22 (66.7%)	105 (82.7%)		
Diabetes				
• Yes	7 (21.2%)	14 (11.0%)	2.17 [0.83–6.12]	0.123
• No	26 (78.8%)	113 (89.0%)		
Other (cardiovascular, lung, kidney diseases, infection) ^a^				
• Yes	4 (12.1%)	11 (8.7%)	1.50 [0.44–5.04]	0.516
• No	28 (84.8%)	115 (90.5%)		
• Missing/unknown ^c^	1 (3.0%)	1 (0.8%)		
Additional supplement intake				
• Yes	14 (42.5%)	53 (41.7%)	1.06 [0.48–2.31]	0.890
• No	18 (54.5%)	72 (56.7%)		
• Missing/unknown ^c^	1 (3.0%)	2 (1.6%)		
History of cancer in family ^a^				
• Yes	15 (45.5%)	58 (45.7%)	1.01 [0.47–2.18]	0.982
• No	18 (54.5%)	69 (54.3%)		
**Arm morbidities symptoms**^a^ Heaviness & tightness of the chest				
• Yes	10 (30.3%)	37 (29.1%)	1.05 [0.45–2.41]	0.916
• No	23 (69.7%)	89 (70.1%)		
• Missing/unknown ^c^	0 (0.0%)	1 (0.8%)		
Hardness & difficulties in finding t-shirts that fits				
• Yes	22 (66.7%)	38 (29.9%)	4.63 [2.05–10.49]	<0.001 **
• No	11 (33.3%)	88 (69.3%)		
• Missing/unknown ^c^	0 (0.0%)	1 (0.8%)		
Pain at the arm				
• Yes	21 (63.7%)	61 (48.0%)	2.00 [0.89–4.50]	0.089
• No	11 (33.3%)	64 (50.4%)		
• Missing/unknown ^c^	0 (0.0%)	2 (1.6%)		
Swelling of the arm				
• Yes	31 (93.9%)	22 (17.3%)	73.2 [16.3–329.6]	<0.001 **
• No	2 (6.1%)	104 (81.9%)		
• Missing/unknown ^c^	0 (0.0%)	1 (1.8%)		

^a^ *p* value was calculated based on test of association using the Pearson’s chi-squared test, ^b^ *p* value obtained from Fisher’s exact test, ^c^ *p* value does not include missing data, ^d^ *p* value and odd ratio obtained from univariate logistic regression. *p* value in bold represents statistically significant difference observed between the two groups tested where * *p* < 0.05 and ** *p* < 0.001. ER = estrogen receptor; PR = progesterone receptor; HER2 = human epidermal growth factor receptor 2.

**Table 4:** Binary logistic regression analysis for the factors associated with BCRL.

Variable	β	S.E	Wald	*p* Value	OR	95% CI
Higher BMI (≥25 kg/m^2^)	0.898	0.449	3.300	0.069	2.45	[0.95–6.46]
Lumpectomy & mastectomy	1.763	0.836	4.489	0.034	5.83	[1.14–29.78]
Hypertension	0.882	0.467	3.572	0.059	2.41	[0.99–6.03]
Post-surgery rehabilitation (<2)	0.806	0.439	3.365	0.067	2.24	[0.95–5.23]

Logistic regression using enter fashion. Variables were categorized as BMI (≥25 kg/m^2^, <25 kg/m^2^), lumpectomy and mastectomy (yes/no), hypertension (yes/no) and post-surgery rehabilitation treatment (one-type rehabilitation treatment/taking two or more rehabilitation treatments). BMI = body mass index; S.E. = standard error; OR = odds ratio; CI = confidence interval.

**Table 5:** Univariate analysis of factors associated with onset of BCRL.

Characteristics	Early-Onset (*n* = 16)	Late-Onset (*n* = 17)	OR [95% CI]	*p* Value
Age during recruitment ^a^	51.2 ± 8.6	52.2 ± 7.9		0.719
BMI, (kg/m^2^) ^a^	31.0 ± 5.1	29.1 ± 5.0		0.294
FACT-B score ^a^	95.9 ± 21.7	111.4 ± 19.4		0.040 *
DASH score ^a^	42.0 ± 18.8	32.6 ± 18.3		0.153
No. of lymph nodes removed ^a^, (*n* = 28)	19.0 ± 6.5	12.2 ± 8.9		0.031 *
**Types of surgery ^b^**				
• Lumpectomy	2	4	0.46 [0.07–2.97]	0.656
• Mastectomy	12	11	1.63 [0.36–7.38]	0.708
• Mastectomy & lumpectomy	2	2	1.07 [0.13–8.67]	1.000
**Types of treatment ^b^**				
• Chemotherapy	10	15	0.22 [0.37–1.33]	0.118
• Radiotherapy	10	14	0.36 [0.07–1.78]	0.259
• Hormonal therapy	12	15	0.40 [0.06–2.57]	0.398
**Co-morbidities**				
• Hypertension ^c^	6	5	1.44 [0.34–6.16]	0.622
• Diabetes ^b^	6	1	9.60 [1.00–91.96]	0.039 *

^a^ *p* value was calculated using independent t-test, ^b^ *p* value obtained from Fisher’s exact test, ^c^ *p* value obtained from Pearson’s chi-squared test. * indicates *p* < 0.05. BMI = body mass index; FACT-B = Functional Assessment of Cancer Therapy -Breast; DASH = Disabilities of Arm, Shoulder and Hand; OR = odd ratio; CI = confidence interval. *p* value in bold represents statistically significant difference observed between the two groups tested.

**Table 6:** Comparison of arm circumference measurement between affected and unaffected arms of women in lymphedema group (*n* = 33).

Point of Measure (cm)	Affected Arm Mean ± SD	Unaffected Arm Mean ± SD	Mean Difference Mean ± SD	*p* Value
Metacarpo-phalangeal	18.17 ± 1.37	17.73 ± 1.09	0.44 ± 0.96	0.014 *
Wrist	16.52 ± 2.00	15.70 ± 1.24	0.82 ± 1.43	0.003 **
Forearm (10 cm below epicondyle)	25.03 ± 3.86	22.97 ± 2.91	2.07 ± 2.48	<0.001 ***
Upper arm (15 cm above epicondyle)	33.37 ± 4.32	32.03 ± 4.16	1.34 ± 1.91	<0.001 ***

*p* value was calculated based on paired *t*-test. * *p* value < 0.05, ** *p* value < 0.01 and *** *p* < 0.001. SD; standard deviation.

## Data Availability

The datasets generated during the current study are not available publicly due to domestic regulation of the institution. However, they are available upon request to the corresponding author.
